# Vascular anatomy of the right colon: A cadaveric study in a Vietnamese population with surgical implications

**DOI:** 10.1371/journal.pone.0358855

**Published:** 2026-09-24

**Authors:** Duc Minh La, Viet Van Ung, Vu Hoang Nguyen, HuyBang Nguyen

**Affiliations:** 1 Department of Anatomy, School of Medicine, University of Medicine and Pharmacy at Ho Chi Minh City (UMP), Ho Chi Minh City, Vietnam; 2 Department of General Surgery, School of Medicine, University of Medicine and Pharmacy at Ho Chi Minh City (UMP), Ho Chi Minh City, Vietnam; AIIMS: All India Institute of Medical Sciences, INDIA

## Abstract

The right colon receives its blood supply from branches of the superior mesenteric artery (SMA), and its venous drainage converges on the superior mesenteric vein (SMV), in part via the gastrocolic trunk (Henle’s trunk). Anatomical variations in these vessels are clinically significant during complete mesocolic excision (CME) with central vascular ligation (CVL) for right-sided colorectal cancer. Population-specific cadaveric data from Vietnam are lacking. A cross-sectional descriptive study was conducted on 31 embalmed adult Vietnamese cadavers (16 female, 15 male; mean age 75.4 ± 11.0 years) at the Department of Anatomy, University of Medicine and Pharmacy at Ho Chi Minh City, between April 2024 and May 2026. After standardized laparotomy, mesenteric dissection was performed from root to periphery. The SMA was left of the SMV in 96.8% of specimens; one variant (3.2%) showed right-of-SMV positioning. The ileocolic artery (ICA) was present in 100% of specimens and always originated from the SMA; 74.2% coursed posterior to the SMV and 25.8% anterior. The right colic artery (RCA) was absent in 35.5%, and when present originated from the SMA (55.0%), middle colic artery (MCA) (40.0%), or ICA (5.0%). The MCA was present in 100% of specimens; a dual-MCA variant was found in 6.5%. The ileocolic vein drained to the SMV in 100% of cases. The gastrocolic trunk (GCT) was present in 71.0% of specimens (absent in 29.0%), with a median length of 11.04 mm (IQR 10.40–12.74 mm). This study provides the first comprehensive cadaveric dataset of right colic vascular anatomy in Vietnamese subjects. Key findings — including the 35.5% absence rate of the RCA, 25.8% anterior ICA crossing, and 29.0% GCT absence — serve as direct intraoperative warning indicators for Vietnamese laparoscopic colorectal surgeons: approximately 1 in 4 patients will have the ICA crossing anterior to the SMV (mandating anterior-surface inspection before peritoneal incision), 1 in 3 will lack the RCA (precluding its use as a reliable landmark), and nearly 1 in 3 will have no GCT (requiring individual tributary ligation at the pancreatic head). These proportions constitute population-specific benchmarks that should inform preoperative planning and intraoperative decision-making during CME-CVL in this setting.

## Introduction

Colorectal cancer (CRC) is among the most common gastrointestinal malignancies worldwide, ranking third in incidence and second in cancer-related mortality globally [1]. Right-sided colon cancer accounts for approximately one-third of all CRC cases, and the burden of CRC is rising in Asia, including Vietnam [1]. Surgery remains the cornerstone of curative treatment, and oncological outcomes depend critically on the quality of the operation [2].

Complete mesocolic excision (CME) with central vascular ligation (CVL), introduced by Hohenberger et al. in 2009 [3], applies the embryological principle of sharp dissection within embryological planes to the colon, analogous to total mesorectal excision for rectal cancer [4]. In a population-based study, CME-CVL was associated with higher 4-year disease-free survival than conventional colon cancer surgery (85.8% vs. 75.9%) [2]. However, technical execution requires intimate knowledge of the vascular anatomy supplying and draining the right colon — anatomy that is notoriously variable.

The right colon is supplied by three main branches of the SMA: the ICA, the RCA, and the right branch of the MCA. Venous drainage returns to the SMV, partly through the GCT (also known as Henle’s trunk), which receives tributaries from the stomach, pancreas, and right colon [5,6]. Injury to the SMV or GCT during mesocolic dissection is a recognized cause of life-threatening hemorrhage [7].

A meta-analysis of 41 studies (n=4,691) by Cirocchi et al. [8] reported the ICA as an almost constant vessel (99.7%) and the RCA as absent in 27.4%. However, the majority of data originate from European, Chinese, Japanese, or Korean populations. Vietnamese-specific cadaveric data are absent from the literature. Given evidence of inter-population anatomical differences in East and Southeast Asia — and the increasing adoption of CME-CVL by Vietnamese colorectal surgeons — population-specific reference data are needed.

In the present study, we aimed to describe the arterial and venous anatomy of the right colon in Vietnamese adult cadavers, with particular focus on features relevant to CME-CVL, including crossing relationships of the ICA with the SMV, presence and origin of the RCA, the number of MCAs, and the presence and length of the GCT.

## Materials and methods

### Study design

A cross-sectional descriptive cadaveric study was conducted in accordance with the Declaration of Helsinki. The study was reviewed and approved by the Institutional Review Board for Biomedical Research, University of Medicine and Pharmacy at Ho Chi Minh City (IRB-VN01002/IORG0008603/FWA00023448; approval No. 520/IRB-UMP, study code 24240-UMP), dated 27 March 2024, under expedited review. All specimens were bodies that had been voluntarily donated for medical education and research; written informed consent for donation and for the use of the body in teaching and research was provided by the donors during life or by their next of kin, in accordance with the body-donation programme of the Department of Anatomy. As the study used only donated cadaveric material and involved no living participants, no additional participant informed consent was required.

This report follows the STROBE (Strengthening the Reporting of Observational Studies in Epidemiology) recommendations for cross-sectional studies; a completed checklist is provided as Supporting Information (S1 Checklist).

### Cadaveric sample

Embalmed cadavers of adult Vietnamese individuals stored at the Department of Anatomy, University of Medicine and Pharmacy at Ho Chi Minh City, were screened between April 2024 and May 2026. Inclusion criteria: Vietnamese adults (≥ 18 years), intact abdomen, no prior dissection of the right colic vasculature. Exclusion criteria: prior abdominal surgery in the right colon region; evidence of malignancy, pancreatic head tumour, or other disease distorting the mesenteric vasculature at time of dissection.

All specimens had been embalmed by routine arterial perfusion with 10% formalin following the standard protocol of university anatomy departments in Vietnam. The interval from death to embalming was less than 8 hours in all specimens, in accordance with the programme’s standard fixation protocol; the subsequent embalming-to-dissection preservation interval is reported per specimen in the Supporting Information. The body-donation programme accepts only bodies following natural death; by departmental policy, bodies are not accepted when death results from trauma (including road-traffic or occupational injury), so deaths in this cohort were predominantly natural and age-related. Forensic autopsy is not performed on donated bodies, and antemortem diagnoses of vascular disease are not systematically available; the potential influence of subclinical atherosclerosis in this elderly cohort is considered in the Limitations. No specimen showed evidence of previous right hemicolectomy or other relevant abdominal surgery at dissection. A complete per-specimen table of sex, age, and preservation interval is provided in the Supporting Information (S1 Dataset).

### Sample size

Sample size was calculated to estimate the proportion of specimens with the RCA as a direct branch of the SMA, using the formula n = Z^2^_1−α/2_ × p × (1 − p)/ d^2^. With p = 0.5 (to maximise the required sample, consistent with a reported range of 30–70%), d = 0.2 (acceptable error), and Z = 1.96 (95% confidence), the minimum required n was 24 cadavers. A 10% allowance for dissection failure yielded a target of 27. Thirty-one cadavers fulfilling inclusion criteria were available over the study period and were all included. Because the a priori calculation adopted the maximum-variance assumption (p = 0.5), the precision actually achieved for the principal proportions was more modest than the nominal ±20% target: with 31 specimens the two-sided 95% confidence interval for a proportion near 0.35 has a half-width of approximately 16 percentage points. Adopting a literature-based prior (RCA absence ≈ 0.27–0.40) would have required a smaller sample and would not have improved this achieved precision. The study is therefore framed as a descriptive, hypothesis-generating characterization of population-specific patterns rather than a precise prevalence estimate; the sample size adequate for a confirmatory estimate is addressed in the Limitations.

### Dissection protocol

Cadavers were positioned supine. A standardized laparotomy was performed using subcostal, transverse iliac crest, and midline incisions; the abdominal flap was reflected to expose the entire peritoneal cavity. The greater omentum and transverse colon were elevated superiorly; the small bowel was displaced to the left to expose the mesenteric root. Dissection proceeded systematically from root to periphery in the following sequence: (1) SMA–SMV axis: full exposure from origin to iliac fossa, recording the positional relationship (SMA left, right, or crossing relative to SMV); (2) ICA: origin from SMA, crossing relationship with SMV (anterior or posterior), length and diameter; (3) RCA: presence, origin (direct from SMA, from MCA, or from ICA), crossing relationship with SMV when originating from SMA, length and caliber; (4) MCA: number, origin, length and caliber; (5) ileocolic vein, right colic vein, superior right colic vein, middle colic vein, and GCT: presence, drainage destination, and GCT length.

### Measurement methodology

Arterial diameter was measured at the point of origin using digital calipers. Because embalmed vessels collapse and cannot be measured as open tubes, each vessel was gently flattened with a Kelly clamp and the maximum transverse width (W) of the collapsed vessel was measured. This width represents half of the circumference of the vessel (W = C/2, where C is the circumference). The external diameter was then calculated as d = C/π = 2W/π (π ≈ 3.14). Two consecutive measurements were taken and averaged. This indirect method for collapsed vessels is consistent with the approach described by Yamaguchi et al. [5] and has been validated for embalmed cadaveric preparations. A potential source of measurement error inherent to this technique is the non-standardization of clamp force: if the Kelly clamp is applied with excessive pressure, the formalin-fixed vessel wall may undergo slight lateral spreading, causing W to be marginally overestimated and the derived diameter to be correspondingly inflated. To minimize this effect, a consistent, light apposition force was maintained throughout dissection, and two consecutive measurements were averaged; nonetheless, readers should be aware that this technique introduces a small, non-quantified systematic bias toward overestimation of vessel diameter compared with direct measurement of perfused, in-vivo vessels. Length was measured from the point of origin to the first branching point along the vessel axis; for curved vessels, a non-elastic thread was placed along the vessel and its length measured. GCT length was measured from the point of confluence of the first two tributaries to the point of entry into the SMV, with three measurements averaged.

All measurements were performed by a single observer (a doctoral candidate) under the direct supervision of a departmental anatomy technician, using a digital caliper with a resolution of 0.01 mm. No calibrated or weight-based clamping device was used; the Kelly clamp was closed to the minimal apposition just sufficient to bring the collapsed vessel walls into contact without visible distortion of the fixed wall. To evaluate intra-observer repeatability, every continuous variable was measured on two separate sessions two weeks apart; at the second session the observer was blinded to the values recorded at the first. The mean of the two sessions was used for all subsequent analyses.

### Statistical analysis

Data were entered into Microsoft Excel 2019 and analysed with SPSS v22.0 (IBM Corp., Armonk, NY, USA). Categorical variables are presented as frequency (n) and proportion (%). Continuous variables were assessed for normality using the Shapiro–Wilk test. Normally distributed variables are presented as mean ± standard deviation (SD) with 95% confidence intervals (95% CI); non-normally distributed variables are presented as median with interquartile range (IQR) and range. For proportions, 95% CIs were calculated using the Wilson score method. Between-group comparisons used Fisher’s exact test (categorical), the independent-samples t-test (continuous, normally distributed) or the Mann–Whitney U test (continuous, non-normal).

Intra-observer repeatability of the two measurement sessions was quantified using the intraclass correlation coefficient (two-way mixed effects, absolute agreement, single measurement; ICC(3,1)) with 95% confidence intervals, interpreted using conventional thresholds (values>0.90 indicating excellent reliability); the within-subject coefficient of variation; the paired t-test for systematic between-session bias; and Bland–Altman analysis with the mean difference (bias), 95% limits of agreement, and the repeatability coefficient (1.96 × √2 × within-subject SD). To place the observed proportions in the context of prior reports, our principal categorical findings were compared with published Vietnamese and pooled international data using Fisher’s exact test for independent single-centre samples and the exact one-sample binomial test against meta-analytic pooled estimates; these between-study comparisons are interpreted as exploratory given methodological heterogeneity.

## Results

### Cadaveric sample characteristics

Thirty-one cadavers were included: 16 female (51.6%) and 15 male (48.4%). Mean age was 75.4 ± 11.0 years (range 56–96); no statistically significant difference in mean age was found between sexes (male 75.7 ± 8.8 vs. female 75.2 ± 13.0 years; p = 0.906). Demographic data are summarised in Table 1.

**Table 1:** Sample demographic characteristics.

Characteristic	Classification	n	Proportion (%)
Sex	Male	15	48.4
	Female	16	51.6
	Total	31	100.0
Age group (years)	< 60	3	9.7
	60–69	7	22.6
	70–79	8	25.8
	≥ 80	13	41.9
Age (years)	Mean ± SD	75.4 ± 11.0	
	Range	56–96	

### Superior mesenteric artery — Positional relationship with SMV

In 30 of 31 specimens (96.8%), the SMA lay to the left of the SMV throughout its course — the typical configuration described in standard anatomical texts. One specimen (3.2%) demonstrated the SMA positioned to the right of the SMV at the level of the third part of the duodenum, representing a rare variant (Table 2); in this specimen the SMV lay directly posterior to the SMA at the level of the pancreatic head and crossed anteriorly to its left more caudally. No other specimen showed a crossing pattern. The mean SMA diameter was 7.80 ± 1.90 mm (95% CI 7.13–8.47; range 4.48–12.46 mm; Shapiro–Wilk p = 0.310).

**Table 2:** Positional relationship of the SMA with the SMV.

Positional relationship	n	Proportion (%)
SMA left of SMV (typical)	30	96.8
SMA right of SMV (variant)	1	3.2
Total	31	100.0

The variant specimen is described in the Results (SMA–SMV relationship) and in the Limitations.

### Ileocolic artery

**Fig 1:**
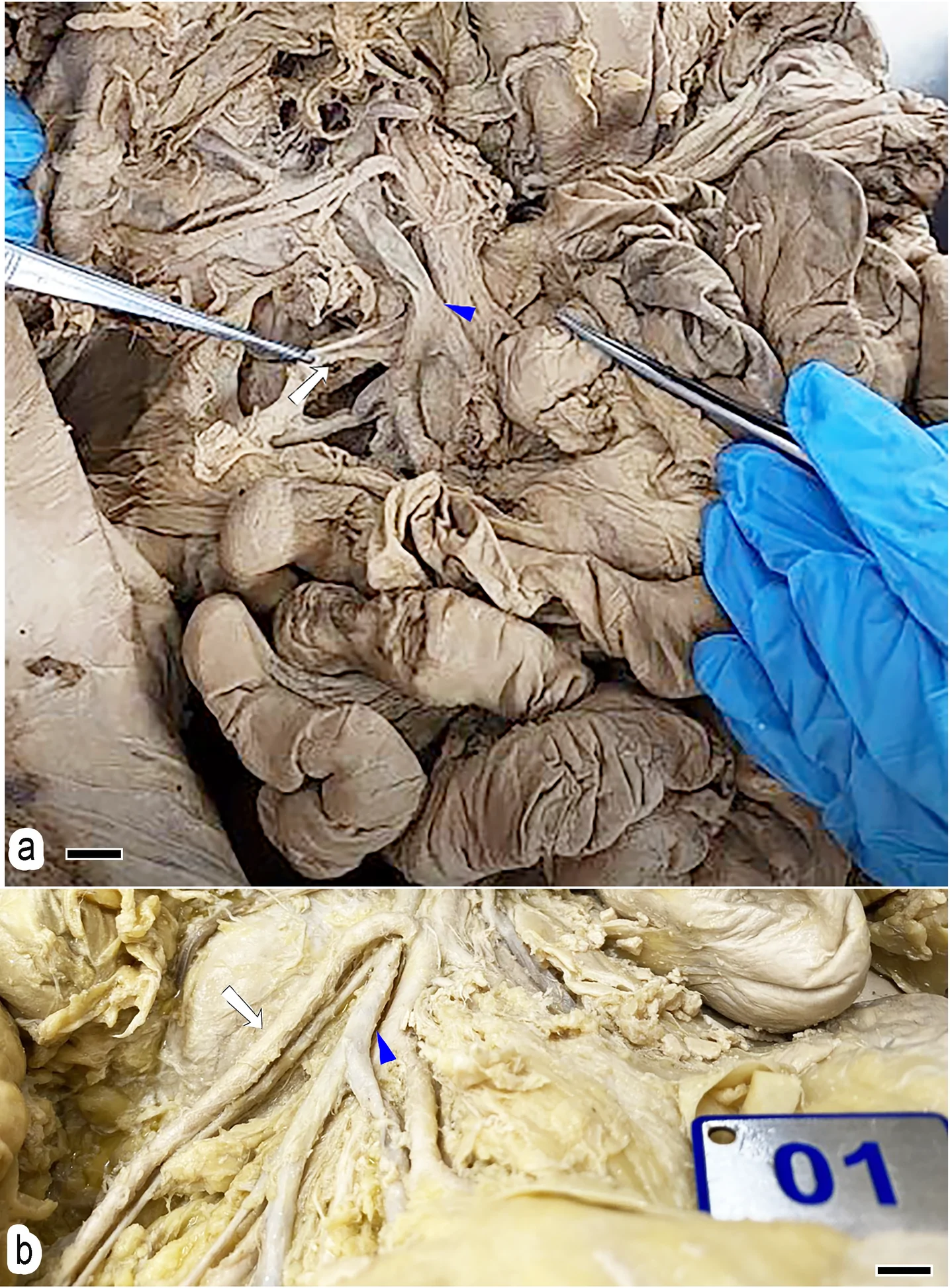
Representative cadaveric dissection photograph illustrating the crossing relationship of the ileocolic artery (ICA) with the superior mesenteric vein (SMV). (a) ICA (white arrow) coursing posterior to the SMV (blue arrow head) (typical pattern, n = 23, 74.2%). (b) ICA (white arrow) crossing anterior to the SMV (blue arrow head) (variant pattern, n = 8, 25.8%). ICA, ileocolic artery; SMA, superior mesenteric artery; SMV, superior mesenteric vein. Scale bar: 1 cm.

The ICA was identified in all 31 specimens (100%), always as a direct branch of the SMA. No accessory or absent ICA was observed. Crossing relationship with the SMV: in 23 specimens (74.2%; 95% CI 56.8–86.3%), the ICA coursed posterior to the SMV; in 8 (25.8%; 95% CI 13.7–43.2%), the ICA crossed anterior to the SMV (Table 3 and Fig 1). Dimensional data are presented in Table 4. All three size variables showed normal distribution (Shapiro–Wilk p > 0.05). Mean ICA length was 60.33 ± 15.97 mm (range 23.58–94.18 mm); mean diameter at origin 4.84 ± 1.23 mm; mean distal diameter 4.50 ± 1.05 mm.

**Table 3:** Crossing relationship of the ICA with the SMV.

Crossing relationship	n	Proportion (%)	95% CI (Wilson)
ICA posterior to SMV	23	74.2	56.8–86.3%
ICA anterior to SMV	8	25.8	13.7–43.2%
Total	31	100.0	

**Table 4:** Dimensional characteristics of the ileocolic artery.

Variable	n	Mean ± SD (mm)	95% CI (mm)
Total length	31	60.33 ± 15.97	54.71–65.96
Diameter at origin	31	4.84 ± 1.23	4.41–5.27
Distal diameter	31	4.50 ± 1.05	4.13–4.87

### Right colic artery

**Fig 2:**
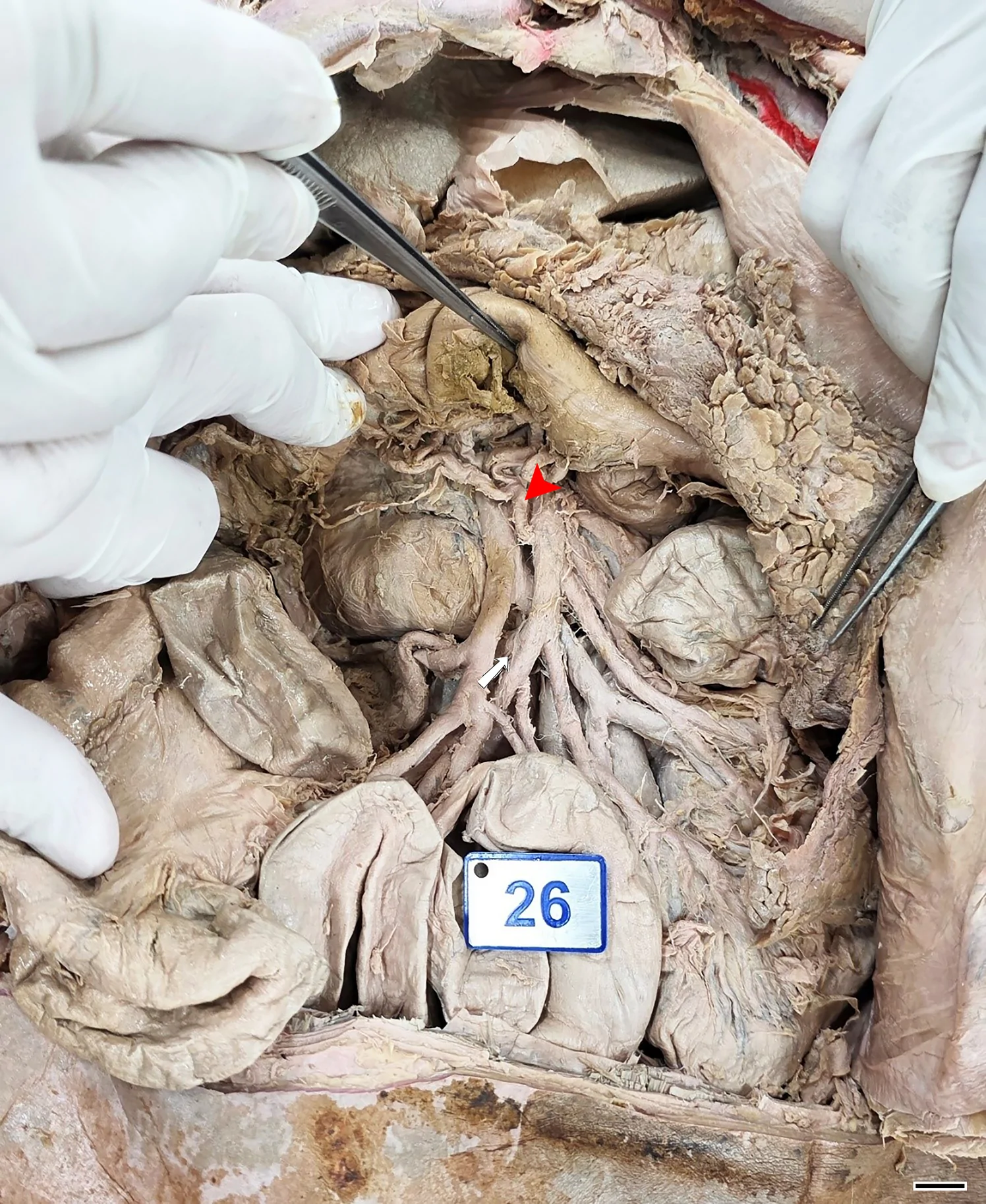
Cadaveric dissection photograph of a specimen in which the right colic artery (RCA) is absent. The ileocolic artery (ICA, white arrow) and middle colic artery (MCA, red arrowhead) are identified; no RCA branch is seen arising from the SMA, MCA, or ICA. This pattern (RCA absent, n = 11, 35.5%) illustrates the need to avoid assuming the RCA as a constant landmark during right hemicolectomy with CME-CVL. Scale bar: 1 cm.

The RCA was absent in 11 of 31 specimens (35.5%; 95% CI 21.1–53.1%). Among the 20 specimens with an RCA, origin was from the SMA in 11 (55.0%), from the MCA in 8 (40.0%), and from the ICA in 1 (5.0%) (Table 5 and Fig 2). Of the 11 specimens in which the RCA originated directly from the SMA, 10 coursed anterior to the SMV (90.9%; 95% CI 62.3–98.4%) and one posterior (9.1%; 95% CI 1.6–37.7%). RCAs originating from the MCA or ICA did not cross the SMV. Fisher’s exact test comparing the anterior-crossing proportion between the ICA and SMA-originating RCA yielded OR = 0.03, p = 0.0003, confirming that the SMA-originating RCA predominantly runs anterior to the SMV, whereas the ICA predominantly runs posterior.

**Table 5:** Presence and origin of the right colic artery (n = 31).

Finding	n	Proportion (%)
Absent	11 (of 31)	35.5
Present — from SMA directly	11 (of 20)	55.0*
Present — from MCA	8 (of 20)	40.0*
Present — from ICA	1 (of 20)	5.0*

* Proportions calculated within the subgroup with RCA present (n = 20).

Mean RCA length was 43.99 ± 13.97 mm (range 23.83–69.57 mm; n = 19; Shapiro–Wilk p = 0.226). One specimen with an RCA present was excluded from the length measurement due to damage to the vessel during initial dissection, rendering accurate measurement impossible; the remaining morphological data (origin, crossing relationship, and diameters) were recorded from that specimen. The diameter at origin was non-normally distributed (Shapiro–Wilk p = 0.008), with median 3.56 mm (IQR 3.04–4.50; range 2.12–8.54 mm). Mean distal diameter was 3.77 ± 1.59 mm (Shapiro–Wilk p = 0.062).

### Middle colic artery

All 31 specimens (100%) had at least one MCA. A single MCA was found in 29 specimens (93.5%; 95% CI 79.3–98.2%); two specimens (6.5%; 95% CI 1.8–20.7%) had a dual MCA arising as separate trunks from the SMA (Table 6). For the primary MCA: length showed non-normal distribution (Shapiro–Wilk p = 0.002), with median 27.04 mm (IQR 20.08–45.09; range 13.46–84.66 mm). Mean diameter at origin was 4.30 ± 1.22 mm (95% CI 3.87–4.73 mm); mean distal diameter 4.31 ± 1.18 mm (95% CI 3.90–4.73 mm). In the two specimens with dual MCAs, the accessory vessels measured: lengths 55.59 mm and 60.94 mm; diameters at origin 3.58 mm and 1.21 mm, respectively.

**Table 6:** Number of middle colic arteries.

Number of MCAs	n	Proportion (%)	95% CI (Wilson)
Single MCA	29	93.5	79.3–98.2%
Dual MCA (variant)	2	6.5	1.8–20.7%
Total	31	100.0	

### Venous anatomy of the right colon

An overview of venous drainage patterns is provided in Table 7. The ileocolic vein drained directly to the SMV in 100% of specimens. The right colic vein was absent in 11 specimens (35.5%). Among the 20 with a right colic vein, drainage was to the GCT in 11 (55.0%), to the SMV directly in 8 (40.0%), and to the ileocolic vein in 1 (5.0%). The superior right colic vein (a vessel without an accompanying artery, draining the hepatic flexure and superior ascending colon) was absent in 23 specimens (74.2%); when present (8 specimens, 25.8%), it always drained to the GCT (100%). The middle colic vein was present in all specimens; 30 of 31 (96.8%; 95% CI 83.8–99.4%) drained to the SMV; in one specimen the drainage destination could not be recorded because the middle colic vein was inadvertently lacerated near its termination during mobilization of the transverse mesocolon, precluding reliable identification of its terminal drainage site.

**Table 7:** Summary of venous drainage patterns of the right colon (n = 31).

Vessel	Presence (%)	Drainage destination
Ileocolic vein (ICV)	100%	SMV (100%)
Right colic vein (RCV)	64.5% (20/31)	GCT 55.0%, SMV 40.0%, ICV 5.0%
Superior right colic vein (SRCV)	25.8% (8/31)	GCT 100%
Middle colic vein (MCV)	100%	SMV 96.8%, unrecorded 3.2%
Gastrocolic trunk (GCT)	71.0% (22/31)	SMV (all)

### Gastrocolic trunk

**Fig 3:**
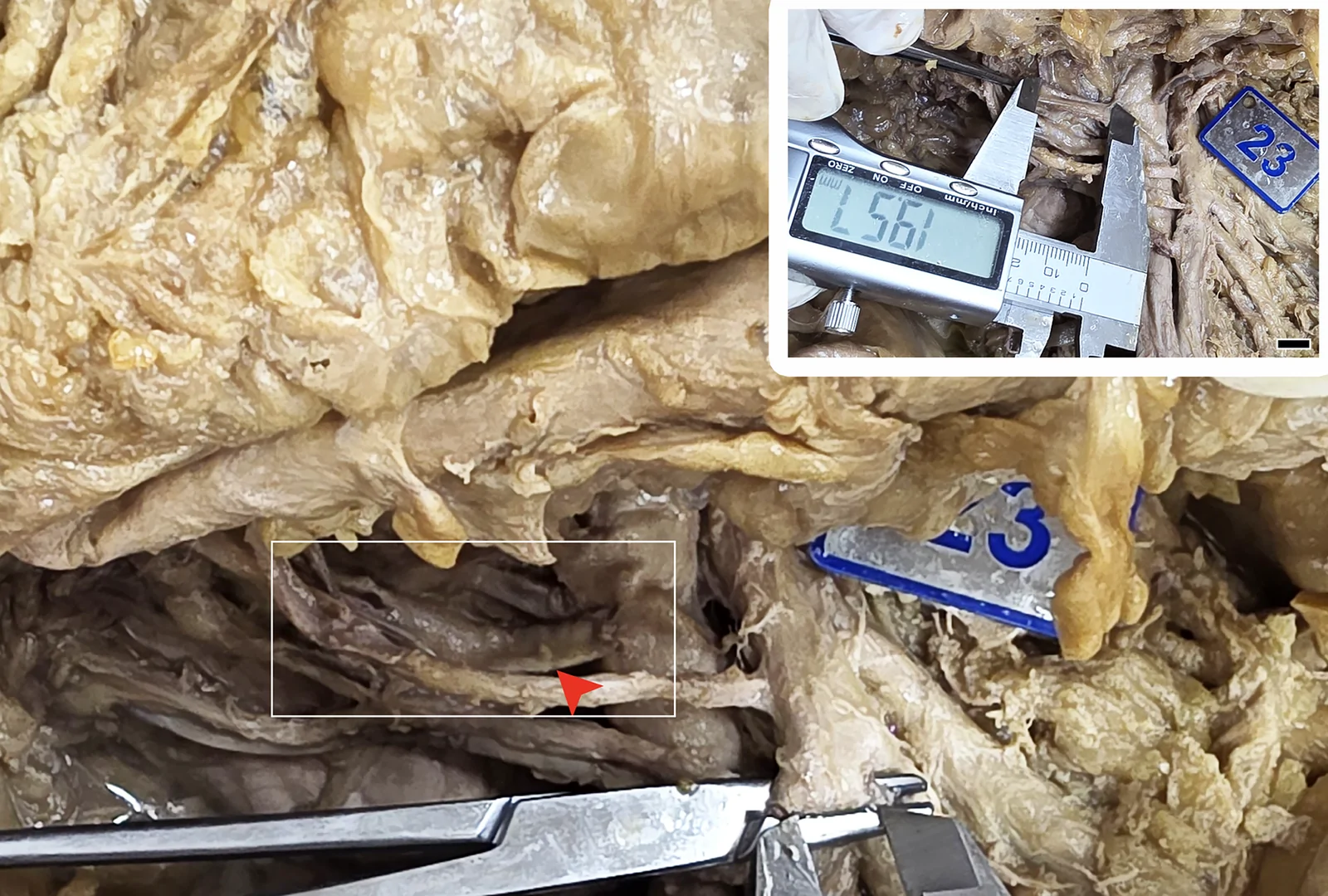
Cadaveric dissection photograph demonstrating the gastrocolic trunk (GCT, Henle’s trunk, red arrow head). GCT present (n = 22, 71.0%) showing confluence of the right gastroepiploic vein and superior right colic vein before entering the SMV. Scale bar: 1 cm.

The GCT was identified in 22 of 31 specimens (71.0%; 95% CI 53.4–83.9%); it was absent in 9 (29.0%; 95% CI 16.1–46.6%). GCT length showed non-normal distribution (Shapiro–Wilk p < 0.001) with median 11.04 mm (IQR 10.40–12.74; range 9.09–19.57 mm) (Table 8 and Fig 3). When absent, the component veins drained independently to the SMV.

**Table 8:** Length of the gastrocolic trunk (GCT) in specimens where it was present (n = 22).

Variable	n	Median (mm)	IQR [Q1–Q3] (mm)	Range (mm)
GCT length	22	11.04	10.40–12.74	9.09–19.57

### Measurement reliability

Intra-observer repeatability of the paired measurement sessions was excellent for every continuous variable. The intraclass correlation coefficient (ICC(3,1)) ranged from 0.990 to 0.999, and the within-subject coefficient of variation from 1.0% to 2.3% (Table 9). Bland–Altman analysis showed no clinically meaningful systematic difference between the two sessions: mean biases were within ±0.06 mm for all vessel diameters (all paired-t p > 0.05) and the repeatability coefficients for diameters were ≤ 0.34 mm. For lengths, the only variable reaching nominal significance was RCA length (mean bias +0.31 mm on a mean of 44 mm; p = 0.049), a difference of no anatomical or surgical consequence. Because all measurements were made by a single observer, inter-observer reliability could not be assessed (see Limitations).

**Table 9:** Intra-observer measurement reliability across two sessions two weeks apart (ICC, within-subject CV, and Bland–Altman statistics). CV, coefficient of variation; RC, repeatability coefficient; bias = mean of (session 1 − session 2). †Nominal p = 0.049; clinically negligible.

Structure	Variable	n	ICC(3,1) (95% CI)	CV (%)	Bias (mm)	RC (mm)
SMA	Diameter	30	0.997 (0.993–0.998)	1.39	−0.03	0.29
ICA	Length	31	0.997 (0.994–0.999)	1.35	−0.06	2.41
ICA	Diameter (origin)	31	0.990 (0.979–0.995)	2.00	−0.01	0.34
ICA	Diameter (distal)	30	0.997 (0.993–0.998)	1.37	−0.01	0.16
RCA	Length	19	0.999 (0.997–1.000)	1.00	+0.31†	1.38
RCA	Diameter (origin)	19	0.999 (0.997–1.000)	1.74	+0.01	0.16
RCA	Diameter (distal)	19	0.998 (0.994–0.999)	2.28	+0.02	0.21
MCA	Length	31	0.999 (0.998–1.000)	1.39	+0.06	1.47
MCA	Diameter (origin)	31	0.998 (0.995–0.999)	1.89	−0.01	0.16
MCA	Diameter (distal)	31	0.996 (0.993–0.998)	1.98	−0.04	0.20
GCT	Length	22	0.996 (0.990–0.998)	1.41	+0.03	0.49

## Discussion

### SMA–SMV relationship

The SMA was positioned to the left of the SMV in 96.8% of specimens, consistent with classical anatomical descriptions and with the CT study of Zerin and DiPietro [9], in which the SMV lay anterior and to the right of the SMA in 88.8% of patients. One specimen (3.2%) demonstrated the SMA to the right of the SMV. Although SMA–SMV inversion is classically associated with intestinal malrotation, it may also occur with normal midgut rotation [9]; in this specimen the duodenojejunal flexure was normally positioned, indicating an isolated vascular variant, which has been reported in detail elsewhere [10]. Such a variant carries important operative implications: in a medial-to-lateral laparoscopic approach, the SMA–SMV relationship is used to orient the dissection plane; if the surgeon assumes the typical left-of-SMV position, the rare rightward variant may result in inadvertent vascular injury.

The mean SMA diameter of 7.80 mm (range 4.48–12.46 mm) is consistent with reported ranges of 6–10 mm [11]. The wide range underscores the need for preoperative CT assessment of vessel caliber when planning instrument selection for CME-CVL.

### Ileocolic artery

The ICA was present in 100% of specimens — consistent with pooled rates of 99.7–99.8% [8,12]. This constancy makes the ICA the most reliable anatomical landmark for initiating right mesocolic dissection in the medial-to-lateral approach [3].

The anterior ICA crossing proportion of 25.8% in the present study is lower than the 50% reported by Lee et al. [13] and Murono et al. [14] using laparoscopy and CT respectively, but is broadly consistent with cadaveric studies: Shatari et al. [15] reported 33.3% and Ignjatovic et al. [16] 36.7%. Of particular relevance is the 35.4% (17/48) anterior rate reported in a Vietnamese laparoscopic series [17], which aligns directionally with the 25.8% obtained here (Table 10). The practical consequence of this roughly 1-in-4 frequency is summarized in the Surgical implications and take-home messages, rather than restated here.

**Table 10:** Comparison of ICA–SMV crossing relationships across studies.

Author (year)	Method	Anterior (%)	Posterior (%)
Shatari et al. (2003)	Cadaveric dissection	33.3	66.7
Ignjatovic et al. (2007)	Fresh cadavers	36.7	63.3
Lee et al. (2016)	Laparoscopy	50.0	50.0
Murono et al. (2016)	CT angiography	50.6	49.4
Nguyen Hoang Bac et al. (2010)	Laparoscopy, Vietnamese	35.4	64.6
Present study (2026)	Cadaveric dissection, Vietnamese	25.8	74.2

Studies used different methods (cadaveric dissection, laparoscopic intraoperative identification, and CT angiography); direct numerical comparisons should be interpreted with caution given differences in methodology, population, and vessel identification criteria. Statistical comparison (this study vs. Nguyen Hoang Bac et al., 2010): anterior crossing 25.8% (8/31) vs. 35.4% (17/48), Fisher’s exact p = 0.46 (not significant); pooled Vietnamese estimate 31.6% (25/79; 95% CI 22.4–42.5%).

ICA dimensions (length 60.33 ± 15.97 mm; origin diameter 4.84 ± 1.23 mm) are consistent with published cadaveric data [18]. The wide length range is clinically relevant, as a very short ICA leaves limited dissection space in laparoscopic approaches.

### Right colic artery

The RCA was absent in 35.5% of specimens — within the range of pooled estimates of 27.4% (Cirocchi et al. [8]) to 39.9% (Negoi et al. [12]) (Table 11) — and consistent with the 38.6% absence rate reported in a Vietnamese laparoscopic series [19]. This relatively high absence rate confirms that the RCA cannot be assumed to be a constant landmark in operative planning; approximately 1 in 3 Vietnamese patients will lack it. When present, the most frequent origin was from the SMA (55.0%), followed by the MCA (40.0%). The latter proportion is higher than the pooled estimate of 15.4% reported by Negoi et al. [12], although close to the 36% reported by Haywood et al. [18]; this may reflect genuine population differences or the small sample size.

**Table 11:** Presence and origin of the RCA: comparison across studies.

Author (year)	Method	Absent (%)	From SMA (%)	From MCA (%)
Haywood et al. (2016)	Fresh cadavers	8.0	32.0	36.0
Negoi et al. (2018)	Meta-analysis	39.9	70.8†	15.4†
Cirocchi et al. (2021)	Meta-analysis	27.4	—	17.7‡
Present study (2026)	Cadaveric, Vietnamese	35.5	55.0*	40.0*

* Proportions within subgroup with RCA present (n = 20). † Negoi et al.: pooled proportions among specimens with an RCA present. ‡ Cirocchi et al.: pooled prevalence of an RCA sharing a common trunk with the MCA. RCA absence in the present series (35.5%) vs. the Cirocchi et al. (2021) pooled estimate (27.4%): exact one-sample binomial p = 0.32 (not significant; the observed value lies within the pooled meta-analytic interval).

The RCA originating from the SMA crossed anterior to the SMV in 90.9% — consistent with the pooled estimate of 89.4% reported by Negoi et al. [12] — forming a useful discriminating landmark: an arterial structure crossing the anterior SMV surface is far more likely to be the RCA than the ICA (OR = 0.03; p < 0.001). When the RCA originated from the MCA or ICA, no SMV crossing was observed.

### Middle colic artery

The MCA was present in 100% of specimens, consistent with pooled rates of 94.6–96.9% [8,12]. A dual-MCA pattern was found in 6.5%, somewhat lower than the pooled prevalences reported in meta-analyses (supernumerary MCA 11.4% [8]; two MCAs 10.6% [12]). Dual MCAs carry specific risk during extended right hemicolectomy: failure to recognize the accessory MCA may result in an incomplete oncological resection or, conversely, inadvertent devascularization of the remaining transverse colon [20]. The relatively short median primary-MCA length (27.04 mm; range 13.46–84.66 mm) implies that in more than half of specimens the MCA bifurcated within 3 cm of its origin, leaving little room for selective ligation of the right branch without risking the left branch and transverse colon perfusion.

### Venous anatomy and gastrocolic trunk

The ileocolic vein drained to the SMV in all 31 specimens, confirming its value as the most constant venous landmark for CVL. The right colic vein showed the most variable drainage pattern, with 35.5% absence and, when present, three possible destinations — the GCT (55.0%), SMV (40.0%), or ileocolic vein (5.0%). This variability mandates individual intraoperative identification of the right colic vein before ligation, to prevent inadvertent injury to the GCT or SMV.

The superior right colic vein — characteristically unaccompanied by an artery, and defined in the present study as a vein draining the hepatic flexure and superior ascending colon that enters the GCT independently, without an accompanying arterial branch — was present in only 25.8% of specimens, lower than the pooled 73.9% reported by Negoi et al. [12]. This discrepancy likely reflects definitional differences: several studies use ‘right colic vein’ and ‘superior right colic vein’ interchangeably, or define the superior right colic vein as any vein draining the ascending colon regardless of whether it has an arterial counterpart [21]. In the present study, we applied a strict definition requiring the absence of an accompanying artery, which may identify a smaller subset of vessels than less restrictive criteria. When present, the superior right colic vein invariably drained to the GCT; given its thin, unsupported wall and the short GCT length, it is the structure most at risk of avulsion when the transverse mesocolon is mobilized under tension before adequate division [7].

The GCT was present in 71.0% of Vietnamese cadavers, lower than the 86.9–97.9% reported in meta-analyses and large multicentre laparoscopic series [12,22–24] (Table 12). In the present study, the GCT was defined as a common venous trunk formed by the confluence of at least two of the following tributaries — the right gastroepiploic vein, the superior right colic vein, the right colic vein, and/or the anterosuperior pancreaticoduodenal vein — before draining into the anteromedial aspect of the SMV; this definition follows Yamaguchi et al. [5] and is consistent with the criteria used by Gao et al. [6]. The proportion is closer to the 69–77.5% found in earlier cadaveric and CT studies [5,6]. The discrepancy relative to large operative series most likely reflects two compounding factors. First, embalmed cadaveric veins are collapsed and unperfused, reducing their visual contrast against surrounding connective tissue; thin-walled tributaries of the GCT may therefore go unrecognized in the cadaveric setting, yielding an underestimate of true GCT presence. Second, population differences cannot be excluded, as all large series with high GCT rates originate from East Asian (Chinese, Japanese) or European cohorts. Consequently, the 29.0% GCT absence rate in the present series may, if anything, overestimate true GCT absence; nevertheless, surgeons should be prepared for the possibility that up to one in three Vietnamese patients lacks a GCT, necessitating individual ligation of the component veins.

**Table 12:** Gastrocolic trunk (GCT) presence: comparison across studies.

Author (year)	n	Method	GCT present (%)
Gao et al. (2018)	—	Systematic review	69–100
Negoi et al. (2018)	6,090*	Meta-analysis	89.7
Stefura et al. (2018)	2,686*	Systematic review and meta-analysis	86.9
He et al. (2020)	371	Laparoscopy (multicenter)	97.3
He et al. (2022)	583	Laparoscopy (multicenter)	97.9
Present study (2026)	31	Cadaveric dissection, Vietnamese	71.0

*Pooled sample from systematic review/meta-analysis. GCT presence in the present series (71.0%) vs. the Negoi et al. (2018) pooled estimate (89.7%): exact one-sample binomial p=0.003 (statistically significant). This difference may reflect genuine population variation and/or differing definitions of the trunk, and is interpreted cautiously given the sample size.

The median GCT length was 11.04 mm (IQR 10.40–12.74; range 9.09–19.57 mm), consistent with He et al. [23] (mean 8.5 mm; range 2–30 mm). A short GCT (< 10 mm) provides minimal length for safe dissection and clamping; the pancreatic head region must be approached with extreme caution in these cases. In a multicentre study of 583 patients, He et al. [24] found that a shorter GCT was associated with greater GCT bleeding during laparoscopic D3 right colectomy.

### Surgical implications

Taken together, our findings support several practical recommendations for Vietnamese colorectal surgeons performing CME-CVL. First, preoperative CT angiography with 3D reconstruction may be considered to map the SMA–SMV relationship, ICA crossing pattern, RCA presence/origin, MCA number, and GCT configuration before right hemicolectomy with D3 lymphadenectomy; because the present study was cadaveric and did not directly evaluate CT angiography, this is offered as a hypothesis for prospective evaluation rather than as an established requirement. Ogino et al. [25] showed that preoperative CT evaluation of the venous anatomy can inform laparoscopic CME for right colon cancer.

Second, the ICA should be the primary landmark for initiation of medial-to-lateral dissection. Before opening the peritoneum along the SMV, the surgeon must first inspect the anterior SMV surface for a crossing vessel: if present and tracing toward the ileocolic region, it is likely an anteriorly crossing ICA (a configuration observed in 25.8% of specimens); if tracing toward the transverse colon, it more likely represents an SMA-origin RCA, which coursed anterior to the SMV in 90.9% of such cases.

Third, the GCT should not be assumed present. In approximately 29% of cases, the tributaries (e.g., the right gastroepiploic vein and the right colic vein) drain independently. Failure to recognize GCT absence and inadvertent traction on individual tributaries is a leading cause of hemorrhage at the pancreatic head [7,26].

**Take-home messages (graded by strength of supporting evidence)**

**Strong — supported by the present data:** (1) Use the ileocolic vessels as the primary, most constant landmark for initiating medial-to-lateral dissection (ICA and ileocolic vein present in 100% of specimens). (2) Do not assume the right colic artery is present: it was absent in about 1 in 3 specimens. (3) Do not assume a gastrocolic (Henle’s) trunk is present: it was absent in about 1 in 3 specimens, so anticipate independent tributaries at the pancreatic head. (4) Before incising the peritoneum along the SMV, inspect the anterior SMV surface for a crossing vessel — an anteriorly crossing ICA occurs in about 1 in 4 specimens.

**Tentative — hypothesis-generating, not directly tested here:** Routine preoperative CT angiography with 3D reconstruction may aid mapping of these variants, but this cadaveric study did not evaluate CT angiography; the recommendation is offered as a hypothesis for prospective evaluation rather than established practice.

**Fig 4:**
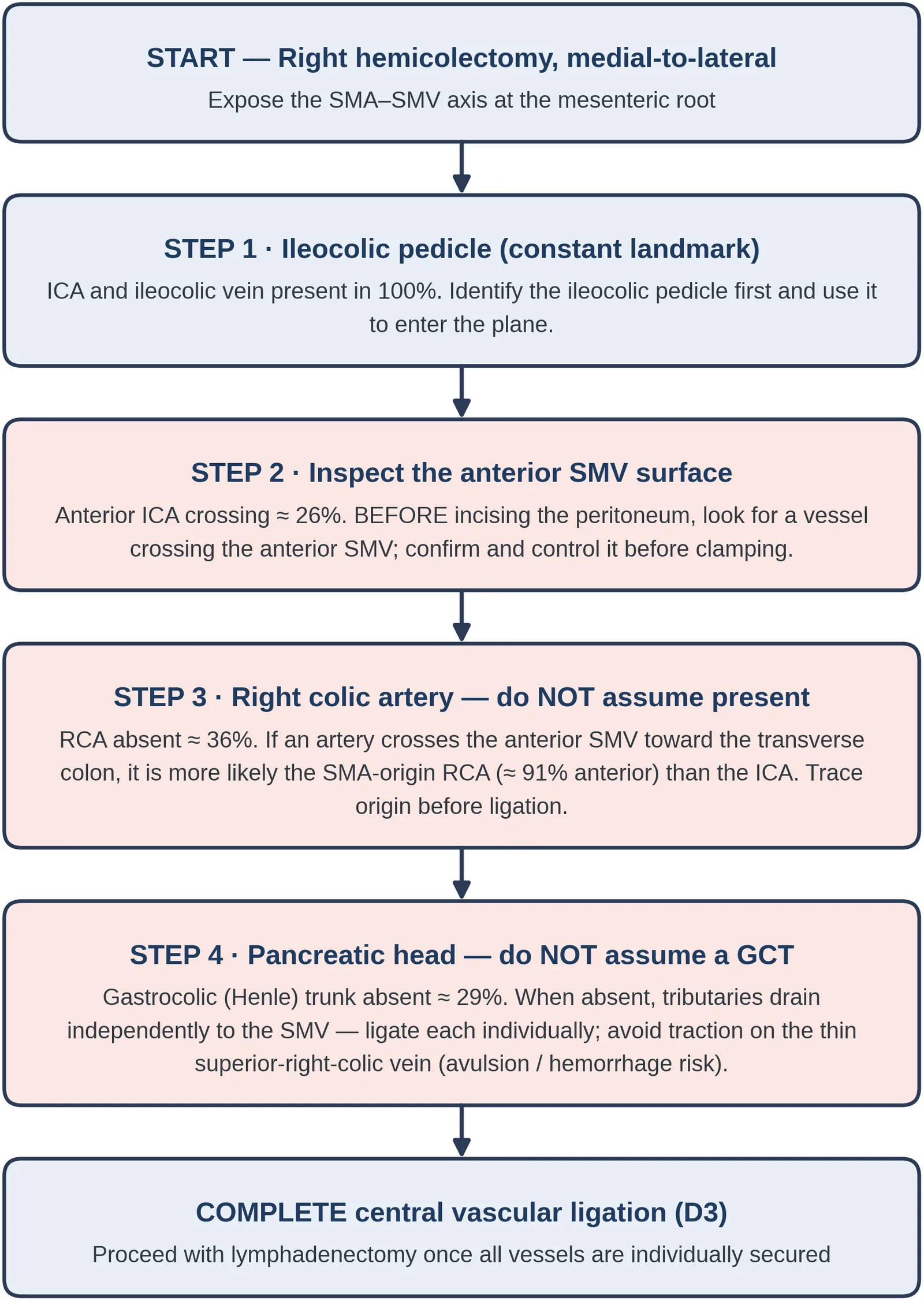
Intraoperative decision aid for CME-CVL of the right colon based on the present findings. Step 1: identify the ileocolic pedicle (constant landmark). Step 2: inspect the anterior SMV surface for a crossing vessel before peritoneal incision (anterior ICA ≈ 26%). Step 3: search for the right colic artery but do not assume its presence (absent ≈ 36%); an artery crossing the anterior SMV toward the transverse colon is more likely the SMA-origin RCA. Step 4: at the pancreatic head, do not assume a gastrocolic trunk (absent ≈ 29%); ligate tributaries individually when it is absent.

An intraoperative decision aid summarizing these points is shown in Fig 4.

### Limitations

This study has several limitations. The sample (n = 31) is small relative to large registry-based CT or operative studies; as a consequence, the 95% CIs for key proportions are wide, particularly for the RCA (95% CI 21.1–53.1% absent) and GCT (95% CI 53.4–83.9% present). The clinical implication of this uncertainty is that the point estimates reported here should be interpreted as preliminary reference values rather than definitive population norms: for example, the true RCA absence rate in the Vietnamese population could plausibly range from approximately 1 in 5–1 in 2 patients, meaning that population-level risk cannot be precisely quantified on the basis of this dataset alone. Using a two-sided 95% CI half-width of 0.10 (i.e., ± 10%) around the observed RCA absence proportion of 35.5%, a future confirmatory study would require a minimum of 88 cadavers (or operative cases) to achieve adequate precision. All measurements were made by a single observer; although intra-observer repeatability was excellent (ICC 0.990–0.999; Table 9), inter-observer reliability could not be assessed, and this remains a limitation. In addition, the single specimen recorded as SMA right of SMV had a more complex course (SMV posterior to the SMA at the pancreatic head, crossing anteriorly to lie to its left at the level of the third part of the duodenum, without intestinal malrotation), which has been reported separately [10]; a simple left/right classification may therefore not capture all SMA–SMV configurations. The use of embalmed cadavers may also alter vascular caliber and venous visibility compared with in-vivo conditions: prolonged formalin fixation tends to reduce measured caliber through tissue shrinkage, whereas the indirect (Kelly-clamp, d = 2W/π) diameter method tends to increase it through possible lateral spreading, so the net effect on absolute diameters is uncertain; importantly, the primary outcomes are categorical branching patterns, which are unaffected by these caliber-related sources of error. Moreover, the study was conducted at a single institution and the cadaveric population was elderly (mean age 75.4 years); some degree of atherosclerotic or degenerative change may have influenced vessel morphology, although branching patterns and topographic relationships are embryologically determined and considered age-stable. Future studies combining cadaveric dissection with CT angiography on a larger sample, or multicentre operative data from Vietnamese surgical units, will be required to confirm and refine the population-specific proportions reported here.

## Conclusions

This cadaveric study provides the first systematic and quantitative description of right colic vascular anatomy in Vietnamese adults. The ICA and ileocolic vein are the most constant structures (100%), whereas the RCA (absent 35.5%) and GCT (absent 29.0%) show the highest variability. An anterior ICA crossing of the SMV occurred in 25.8% of specimens — affecting approximately 1 in 4 patients. These data establish preliminary Vietnamese-specific reference values for preoperative planning and intraoperative decision-making in right hemicolectomy with CME-CVL, and provide a baseline for future prospective studies on vascular anatomy and operative outcomes in this population.

## Supporting information

S1 Dataset Complete anonymized per-cadaver dataset (sex, age, preservation interval, and all categorical and continuous vascular measurements). (XLSX)

S1 Checklist STROBE checklist for cross-sectional studies. (DOCX)
