# Supplementary material for: Vascular anatomy of the right colon: A cadaveric study in a Vietnamese population with surgical implications
Source: PLoS One. 2026 Sep 24;21(9):e0358855. doi: 10.1371/journal.pone.0358855 (PMC13606972; doi:10.1371/journal.pone.0358855)
Supplement: Supplementary file 1 [file pone.0358855.s002.docx]

**S1 Checklist. STROBE statement—checklist of items that should be included in reports of cross-sectional studies.**

*Manuscript: “Vascular anatomy of the right colon: A cadaveric study in a Vietnamese population with surgical implications” (PONE-D-26-28364). Locations refer to the correspondingly named sections of the revised manuscript; final page numbers will follow typesetting.*

| **Section/Topic** | **Item** | **Recommendation** | **Reported in revised manuscript** |
| --- | --- | --- | --- |
| ***Title and abstract*** | | | |
| Title and abstract | 1 | (a) Indicate the study’s design with a commonly used term in the title or the abstract. | Title; Abstract (“cross-sectional descriptive study … 31 embalmed adult Vietnamese cadavers”) |
|  | 1 | (b) Provide in the abstract an informative and balanced summary of what was done and what was found. | Abstract |
| ***Introduction*** | | | |
| Background/rationale | 2 | Explain the scientific background and rationale for the investigation being reported. | Introduction, paragraphs 1–4 |
| Objectives | 3 | State specific objectives, including any prespecified hypotheses. | Introduction, final paragraph (“we aimed to describe the arterial and venous anatomy of the right colon …”) |
| ***Methods*** | | | |
| Study design | 4 | Present key elements of study design early in the paper. | Methods – Study design |
| Setting | 5 | Describe the setting, locations, and relevant dates, including periods of recruitment, exposure, follow-up, and data collection. | Methods – Cadaveric sample (Department of Anatomy, UMP-HCM; April 2024–May 2026); Study design |
| Participants | 6 | (a) Give the eligibility criteria, and the sources and methods of selection of participants. | Methods – Cadaveric sample (inclusion/exclusion criteria; embalmed cadavers screened) |
| Variables | 7 | Clearly define all outcomes, exposures, predictors, potential confounders, and effect modifiers. Give diagnostic criteria, if applicable. | Methods – Dissection protocol (variables recorded per vessel); definitions of superior right colic vein and gastrocolic trunk in Methods/Discussion |
| Data sources/ measurement | 8 | For each variable of interest, give sources of data and details of methods of assessment (measurement). Describe comparability of assessment methods if more than one group. | Methods – Measurement methodology (digital calipers 0.01 mm; collapsed-vessel formula d = 2W/π; thread method for length) |
| Bias | 9 | Describe any efforts to address potential sources of bias. | Methods – Measurement methodology (clamp-force minimisation; two averaged readings; blinded second session); Statistical analysis (intra-observer reliability); Discussion – Limitations |
| Study size | 10 | Explain how the study size was arrived at. | Methods – Sample size (a priori formula; post-hoc precision); Discussion – Limitations (confirmatory n = 88) |
| Quantitative variables | 11 | Explain how quantitative variables were handled in the analyses. If applicable, describe which groupings were chosen and why. | Methods – Statistical analysis (Shapiro–Wilk; mean±SD vs median[IQR]; Wilson CIs); Results – Table 1 (age groups) |
| Statistical methods | 12 | (a) Describe all statistical methods, including those used to control for confounding. | Methods – Statistical analysis |
|  | 12 | (b) Describe any methods used to examine subgroups and interactions. | Methods – Statistical analysis; Results – Right colic artery (origin subgroups); sex comparison of age |
|  | 12 | (c) Explain how missing data were addressed. | Results – Right colic artery (1 specimen excluded from length, n = 19) and Venous anatomy (1 middle colic vein drainage unrecorded) |
|  | 12 | (d) If applicable, describe analytical methods taking account of sampling strategy. | Not applicable – all eligible specimens available in the study period were included (census); Methods – Sample size |
|  | 12 | (e) Describe any sensitivity analyses. | Methods – Statistical analysis and Results – Measurement reliability (ICC, CV, Bland–Altman; Table 9); exploratory between-study comparisons (Fisher’s exact, one-sample binomial) |
| ***Results*** | | | |
| Participants | 13 | (a) Report numbers of individuals at each stage of the study. | Results – Cadaveric sample characteristics (31 cadavers included) |
|  | 13 | (b) Give reasons for non-participation at each stage. | Methods – Sample size (target 27; the 31 eligible specimens available were all included; no exclusions after enrolment) |
|  | 13 | (c) Consider use of a flow diagram. | Not applicable – single-stage cadaveric census without sequential attrition |
| Descriptive data | 14 | (a) Give characteristics of study participants and information on exposures and potential confounders. | Results – Cadaveric sample characteristics; Table 1; Methods – Cadaveric sample (embalming, natural-death policy) |
|  | 14 | (b) Indicate number of participants with missing data for each variable of interest. | Results – Right colic artery (n = 19/20 for length) and Venous anatomy (middle colic vein drainage unrecorded in 1); S1 Dataset |
| Outcome data | 15 | Report numbers of outcome events or summary measures. | Results – Tables 2–8 (frequencies, proportions, dimensions with 95% CI) |
| Main results | 16 | (a) Give unadjusted estimates and, if applicable, confounder-adjusted estimates and their precision (e.g., 95% CI). | Results (all subsections; Wilson 95% CIs); Fisher’s exact OR for RCA vs ICA anterior crossing |
|  | 16 | (b) Report category boundaries when continuous variables were categorized. | Results – Table 1 (age-group boundaries: <60, 60–69, 70–79, ≥80) |
|  | 16 | (c) If relevant, consider translating estimates of relative risk into absolute risk. | Not applicable (descriptive anatomical proportions); framed as absolute frequencies (“~1 in 3 / ~1 in 4”) in Abstract and Discussion |
| Other analyses | 17 | Report other analyses done—e.g., analyses of subgroups and interactions, and sensitivity analyses. | Results – Measurement reliability (Table 9); Discussion – Tables 10–12 (between-study statistical comparisons; pooled Vietnamese estimate) |
| ***Discussion*** | | | |
| Key results | 18 | Summarise key results with reference to study objectives. | Discussion (opening of each subsection); Conclusions |
| Limitations | 19 | Discuss limitations of the study, taking into account sources of potential bias or imprecision. Discuss both direction and magnitude of any potential bias. | Discussion – Limitations (small n; single observer; embalming/formalin caliber effects with stated direction; age-related atherosclerosis) |
| Interpretation | 20 | Give a cautious overall interpretation of results considering objectives, limitations, multiplicity of analyses, results from similar studies, and other relevant evidence. | Discussion (all subsections); Conclusions |
| Generalisability | 21 | Discuss the generalisability (external validity) of the study results. | Discussion – Limitations (single institution; “preliminary reference values”); Conclusions |
| ***Other information*** | | | |
| Funding | 22 | Give the source of funding and the role of the funders for the present study and, if applicable, for the original study on which the present article is based. | Funding (“This research received no external funding”) |

*An Explanation and Elaboration article discusses each checklist item and is available at www.strobe-statement.org. Information on the STROBE Initiative is available at the same site.*
